# Comparing self-reported and measured hypertension and hypercholesterolaemia at standard and more stringent diagnostic thresholds: the cross-sectional 2010–2015 Busselton Healthy Ageing study

**DOI:** 10.1186/s40885-022-00199-1

**Published:** 2022-06-01

**Authors:** Angela J Burvill, Kevin Murray, Matthew W Knuiman, Joseph Hung

**Affiliations:** 1 Medical School, University of Western Australia, Crawley, WA Australia; 2 School of Population and Global Health, University of Western Australia, Crawley, WA Australia

**Keywords:** Hypertension, Hypercholesterolaemia, Sensitivity and specificity, Self-report, Validation study

## Abstract

**Background:**

Population health behaviour and risk factor surveys most often rely on self-report but there is a lack of studies assessing the validity of self-report using Australian data. This study investigates the sensitivity, specificity and agreement of self-reported hypertension and hypercholesterolaemia with objective measures at standard and more stringent diagnostic thresholds; and factors associated with sensitivity and specificity of self-report at different thresholds.

**Methods:**

This study was a secondary analysis of a representative community-based cross-sectional sample of 5,092 adults, aged 45–69 years, residing in Busselton, Western Australia, surveyed in 2010–2015. Participants completed a self-administered questionnaire. Blood pressure and serum cholesterol levels were measured.

**Results:**

At currently accepted diagnostic thresholds, sensitivities of self-reported hypertension and hypercholesterolaemia were 58.5% and 39.6%, respectively and specificities were >90% for both. Agreement using Cohen’s kappa coefficient was 0.562 and 0.223, respectively. At two higher diagnostic thresholds, sensitivities of self-reported hypertension and hypercholesterolaemia improved by an absolute 14–23% and 15–25%, respectively and specificities remained >85%. Agreement was substantial for hypertension (kappa = 0.682–0.717) and moderate for hypercholesterolaemia (kappa = 0.458–0.533). Variables that were independently associated with higher sensitivity and lower specificity of self-report were largely consistent across thresholds and included increasing age, body mass index, worse self-rated health, diabetes and family history of hypertension.

**Conclusions:**

Self-reported hypertension and hypercholesterolaemia often misclassify individuals’ objective status and underestimate objective prevalences, at standard diagnostic thresholds, which has implications for surveillance studies that rely on self-reported data. Self-reports of hypertension, however, may be reasonable indicators of those with blood pressures ≥160/100 mmHg or those taking anti-hypertensive medications. Self-reported hypercholesterolaemia data should be used with caution at all thresholds.

## Background

Cardiovascular disease (CVD) is a leading cause of death in Australia and worldwide [1]. Major risk factors for CVD include hypertension (HTN) and hypercholesterolaemia (HC) [2]. Monitoring prevalence of risk factors, research on determinants of health and implementation of public health policy aimed at CVD prevention requires accurate CVD risk factor estimates [2].

Population health risk factor surveillance often relies on self-report surveys, for example, data collected through questionnaires [3–5]. Although self-reporting is one of the easiest and most widely used methods of collecting data about individuals’ health and risk factor status, the evidence also suggests that self-reported data should be used with some caution [6]. In Australia, nationally representative biomedical measures became available for the first time since the 1990’s in a sub-sample of the 2011–2013 Australian Health Survey [7].

It is important to know if self-reports provide reasonable estimates of true prevalences, given the reliance on self-reported data. Several international studies with large random population samples aim to validate self-report by comparing self-reported HTN [8–13] or HC [9, 12–15] data with clinically measured (objective) HTN or HC data. Studies generally found that self-reported HTN and HC have low sensitivities (34–56%) and high specificities (≥88%), with overall effects of substantially under-estimating objective prevalences. Reported Cohen’s kappa (κ) coefficients vary between 0.27 and 0.46, which indicate only fair-moderate agreement. Authors thus conclude that self-reported HTN and HC data should be used with caution [9, 14, 16].

There are limited studies that have compared clinically measured HTN or HC with self-report in large random population samples using Australian data. Taylor et al. [17], using 2002–2003 data on a representative population sample of 1537 adults living in South Australia, found major under-reporting of HTN and HC based on self-report estimates, even though kappa coefficients indicate moderate agreement. Peterson et al. [18], using a cross-sectional sample of 7,269 adults aged ≥18 years participating in the 2011–2012 Australian Health Survey, found significant under-reporting of HTN and HC based on self-report and low agreement with objective measures. However, interpretation of their results is difficult due to the lack of treatment (medication) data.

Factors commonly identified in previous studies associated with higher sensitivity of self-report (which is the proportion of those with objective HTN or HC who also self-reported ‘yes’) include increasing age, higher body mass index (BMI), lower self-rated health or a history of CVD [8–15]. Participants with these risk factors may have increased health consciousness and exposure to monitoring programs [10]. Factors associated with specificity are explored less frequently and results are inconsistent [18–20].

Most studies use currently recommended diagnostic criteria to classify HTN or HC, which are: systolic blood pressure (SBP) ≥140 mmHg, diastolic blood pressure (DBP) ≥90 mmHg and/or use of anti-hypertensive medications; and total cholesterol (TC) ≥5.0–5.5 mmol/L and/or use of lipid-lowering medications. These criteria would classify patients with mild HTN or HC and above in the clinical setting [21, 22]. Some studies have investigated comparisons of self-report using higher diagnostic thresholds for HTN or HC [8, 10–12] and have found large absolute improvements in sensitivity (16–27%) with relatively small declines in specificity (1–2%). A limitation of the studies reporting at higher thresholds is that authors report sensitivity and/or specificity only and all, except Natarajan et al. [14], report at one higher threshold only.

The primary aim of this study was therefore to investigate the sensitivity, specificity, predictive accuracy (positive predictive values [PPV] and negative predictive values [NPV]) and agreement of self-reported and objectively measured HTN and HC at standard and at more stringent diagnostic thresholds using high-quality, contemporary Australian data. The secondary aim was to investigate factors associated with sensitivity and specificity of self-report for objective HTN and HC at increasing diagnostic thresholds. The study was a secondary analysis of a cross-sectional survey of middle-aged adults living in Busselton, Western Australia. The representative community-based cohort was surveyed between 2010 and 2015.

## Methods

### Survey setting and participants

A community-based cross-sectional survey of adults born within 1946–1964 residing in Shire (now City) of Busselton, Western Australia, was undertaken from May 2010 to December 2015. Details of the Busselton Healthy Ageing Study (BHAS) protocol have been described elsewhere [23]. This study was a secondary analysis of this survey. All non-institutionalised adults born 1946–1964 (‘baby boomers’) who lived in the Shire and were listed on the 2010 national electoral roll were invited to participate. Enrolment to vote is compulsory in Australia. Participants were invited to the survey centre to complete a self-administered questionnaire and to undergo comprehensive physical assessments. Contact was made with 82% of those eligible and 76% of those contacted participated. This gave an overall participation rate of 62%. Amongst the 5107 participants, 5092 gave blood. The study received ethics approval from The University of Western Australia’s Human Research Ethics Committee (Number RA/4/1/2203) and written informed consent was obtained from each participant.

### Self-reported variables

A “yes” response to the question, “has (your doctor) ever told you that you have high blood pressure (BP)” or “high cholesterol”, defined positive a self-report of HTN or HC respectively.

Participants were asked to “copy the name of each (doctor-prescribed) medicine exactly as it appears on the package”. Anti-hypertensive and lipid-lowering medications were identified by their principal drug indication and confirmed with a cardiologist (JH).

All variables collected by the questionnaire deemed pertinent to the sensitivity and specificity of self-reported HTN or HC were considered for inclusion as covariates in logistic regression models. Major sociodemographic (age, sex, marital status, income level, highest level of education), lifestyle (smoking status, alcohol intake, physical activity levels), CVD risk factor (history of CVD, self-reported diabetes mellitus, family history of HTN) and quality of life indicator (self-rated health, having a long-standing illness) variables were included in the final analysis. Those variables considered, but excluded, were ancestry of participant (almost all were European ancestry) and self-reported high BP or pre-eclampsia during pregnancy (due to the small proportion of participants to whom that question applied). Drinking ≥14 glasses of alcohol per week was defined as excessive alcohol intake, in accordance with Australian guidelines [24]. Physical activity was calculated as (minutes/week of moderate intensity activities) + 2 x (minutes/week of vigorous intensity activity) [25]. Family history of HTN was defined as self-reporting “yes” to the biological mother and/or father “ever having” HTN. History of CVD was defined self-reporting “yes” to having had angina, myocardial infarction (heart attack), transient ischaemic attack, stroke and/or leg claudication.

### Measured variables

BMI (kg/m^2^) was calculated using weight and height measures. BP was measured in all limbs after five minutes of rest using a vascular profiler (Omron VP1000, Kyoto, Japan). The right brachial measure gave BP [23]. Nationally accredited laboratories [23] performed analyses of TC and low density lipoprotein cholesterol (LDL-C) levels on fasting venous blood samples.

Objective HTN was defined as SBP ≥140 mmHg, DBP ≥90 mmHg and/or using anti-hypertensive medications, based on national [21] and international guidelines [26] at the time of data collection. The highest threshold for objective HTN was SBP ≥160 mmHg, DBP ≥100 mmHg and/or anti-hypertensive medications, which would classify moderate HTN and above in the clinical setting [21]; SBP ≥150 mmHg, DBP ≥95 mmHg and/or anti-hypertensive medications represented a middle threshold.

Objective HC was classified as TC ≥5.5 mmol/L, LDL-C ≥3.5 mmol/L and/or use of lipid-lowering medications, based on 2011–2013 Australian National Health Survey cut-off points [22]. The middle threshold of TC ≥6.2 mmol/L, LDL-C ≥4.1 mmol/L and/or lipid-lowering medications was based on United States’ guidelines (2001) at the time [27]. The highest threshold of TC ≥6.5mmol/L, LDL-C ≥4.9 mmol/L and/or lipid-lowering medications was chosen because TC ≥6.5 mmol/L was the cut-off at the time for very high risk of developing coronary artery disease [28] and LDL-C ≥4.9 mmol/L was the cut-off for ‘very high’ LDL-C levels [27].

### Statistical analysis

The program IBM SPSS ver. 25 (IBM Corp., Armonk, NY, USA) was used for all statistical analyses. Descriptive statistics were presented as median and interquartile range for continuous variables and as number (percentage) for categorical variables.

Sensitivity was the proportion of those with objective HTN or HC who self-reported ‘yes’; specificity was the proportion without objective HTN or HC who self-reported ‘no’. PPV was the proportion of those who self-reported ‘yes’ that had objective HTN or HC. NPV was the proportion of those who self-reported ‘no’ that did not have objective HTN or HC. Cohen’s kappa (κ) coefficient, with 95% confidence intervals (CI), was calculated for agreement between self-reported and objective HTN or HC at each selected threshold. Agreement was classified as slight (κ ≤ 0.20), fair (0.21–0.40), moderate (0.41–0.60), substantial (0.61–0.80), or almost perfect (≥0.81) [29].

Odds ratios (OR) were estimated using multivariable logistic regression to identify participant characteristics that independently predicted better sensitivity or specificity of self-report. The initial multivariable logistic regression model for sensitivity included all potential explanatory variables. The final model included all variables except those that were not significant at any threshold of HTN or HC for sensitivity in the initial model. The sensitivity model for HTN or HC was restricted to those with a positive objective measure, where a ‘yes’ self-report was the dependent variable. The specificity model was restricted to those with a negative objective measure, where a ‘no’ self-report was the dependent variable. The final model included all variables except those that were not significant at any threshold for HTN or HC for specificity in the initial model.

## Results

### Participants

Table 1 summarises the sociodemographic and clinical characteristics of the sample. There were 4,876 participants in the analysis, after excluding participants (4.3% of the total sample) who had missing data for any variable included in the study. Their age range was 45 to 69 years, median age was 58 years, 55% were female, 50% had some formal education beyond high school, 10% were current smokers, 73% were overweight or obese, 5% had a history of CVD and 6% self-reported diabetes. This sample had risk characteristics similar to those in the 2011–2013 Australian Health Survey, which collected nationally representative data [30].

**Table 1:** Socio-demographic and clinical characteristics of the Busselton survey sample (*n *= 4,876)

Characteristics	Frequency (%)
Age at enrolment, median (interquartile range) [range]	58.2 (53.2, 63.0) [45.4, 69.8]
Sex	
Male	2,205 (45.2)
Female	2,671 (54.8)
Marital status	
Married or de-facto	4,034 (82.7)
Other	842 (17.3)
Income ($)	
Prefer not to say, none of the above	647 (13.3)
≤20,0000	290 (5.9)
20,001–40,000	800 (16.4)
40,001–60,000	805 (16.5)
60,001–80,000	640 (13.1)
80,001–100,000	582 (11.9)
≥100,001	1,112 (22.8)
Highest level of education	
Secondary school or lower	2,452 (50.3)
Other educational institution	1,473 (30.2)
University	951 (19.5)
Smoking	
Never	2,296 (47.1)
Former	2,099 (43.0)
Current	481 (9.9)
Alcohol, excessive intake	
No	3,515 (72.1)
Yes	1,361 (27.9)
Physical activity (min/wk)	
<150	1,965 (40.3)
≥150	2,911 (59.7)
Body mass index (kg/m^2^)	
<25	1,302 (26.7)
26–29	2,131 (43.7)
≥30	1,443 (29.6)
Self-rated level of health	
Poor or fair	423 (8.7)
Good	1,898 (38.9)
Very good	1,925 (39.5)
Excellent	630 (12.9)
Long-standing illness	
No	3,730 (76.5)
Yes	1,146 (23.5)
Family history of hypertension	
No or don’t know	2,253 (46.2)
Yes	2,623 (53.8)
History of cardiovascular disease	
No	4,635 (95.1)
Yes	241 (4.9)
Diabetes mellitus	
No	4,568 (93.7)
Yes	308 (6.3)

### Hypertension

Table 2 shows that the prevalence of self-reported HTN was 27.9%, which was considerably lower than the 43.1% objective prevalence of HTN based on the standard threshold but closer to objective prevalences at the middle and higher thresholds (31.6% and 26.3%). The prevalence of anti-hypertensive medication usage amongst all participants was 22.9%.

**Table 2:** Comparisons of sensitivity, specificity, agreement and predictive values of hypertension at increasing diagnostic thresholds

Variable	SBP ≥140 mmHg, DBP ≥90 mmHg and/or anti-hypertensive medications			SBP ≥150 mmHg, DBP ≥95 mmHg and/or anti-hypertensive medications			SBP ≥160 mmHg, DBP ≥100 mmHg and/or anti-hypertensive medications		
	OBJ+	OBJ–	Total	OBJ+	OBJ–	Total	OBJ+	OBJ–	Total
SR+	1,231	127	1,358	1,126	232	1,358	1,047	311	1,358
SR–	872	2,646	3,518	417	3,101	3,518	234	3,284	3,518
Total	2,103	2,773	4,876	1,543	3,333	4,876	1,281	3,595	4,876
Prevalence OBJ+ (%)	43.1 (41.7, 44.5)			31.6 (30.3, 33.0)			26.3 (25.0, 27.5)		
Prevalence SR+ (%)	27.9 (26.6, 29.1)			27.9 (26.6, 29.1)			27.9 (26.6, 29.1)		
Sensitivity (%)	58.5 (56.4, 60.6)			73.0 (70.8, 75.2)			81.7 (79.6, 83.8)		
Specificity (%)	95.4 (94.6, 96.2)			93.0 (92.2, 93.9)			91.3 (90.4, 92.3)		
PPV (%)	90.6 (89.1, 92.2)			82.9 (80.9, 84.9)			77.1 (74.9, 79.3)		
NPV (%)	75.2 (73.8, 76.6)			88.1 (87.1, 89.2)			93.3 (92.5, 94.2)		
Kappa (κ)	0.564 (0.540, 0.586)			0.682 (0.660, 0.704)			0.717 (0.695, 0.739)		

*SBP* systolic blood pressure, *DBP* diastolic blood pressure, *OBJ+* positive objective measure, *OBJ–* negative objective measure, *SR+* positive self-reported measure, *SR–* negative self-reported measure, *PPV* positive predictive value, *NPV* negative predictive value

At the standard threshold, sensitivity was 58.5% and NPV was 75.2%, specificity was 95.4% and PPV was 90.6% and there was moderate agreement (κ = 0.564) of self-report with objective measures. In comparison, at the middle threshold, sensitivity (73.0%) and NPV (88.1%) were higher with substantial agreement (κ = 0.682) and only small declines in specificity (93.0%) and PPV (82.9%). At the highest threshold, sensitivity (81.7%), and NPV (93.3%) increased further without much change in specificity (91.3%) or PPV (77.1%) and with substantial agreement (κ = 0.717).

### Hypercholesterolaemia

Table 3 shows that prevalence of self-reported HC was 30.4%, which was considerably lower than the 70.5% objective prevalence of HC at the standard threshold but closer to the prevalences at the two higher thresholds (44.9% and 34.0%). The prevalence of lipid-lowering medication usage in the population was 16.8%.

**Table 3:** Comparisons of sensitivity, specificity, agreement and predictive values of hypercholesterolaemia at increasing diagnostic thresholds

Variable	TC ≥5.5 mmol/L, LDL-C ≥3.5 mmol/L and/or lipid-lowering medications			TC ≥6.2 mmol/L, LDL-C ≥4.1 mmol/L and/or lipid-lowering medications			TC ≥6.5 mmol/L, LDL-C ≥4.9 mmol/L and/or lipid-lowering medications		
	OBJ+	OBJ–	Total	OBJ+	OBJ–	Total	OBJ+	OBJ–	Total
SR+	1,362	122	1,484	1,201	283	1,484	1,073	411	1,484
SR–	2,078	1,314	3,392	986	2,406	3,392	584	2,808	3,392
Total	3,440	1,436	4,876	2,187	2,689	4,876	1,657	3,219	4,876
Prevalence OBJ+ (%)	70.5 (69.3, 71.8)			44.9 (43.5, 46.2)			34.0 (32.7, 35.3)		
Prevalence SR+ (%)	30.4 (29.1, 31.7)			30.4 (29.1, 31.7)			30.4 (29.1, 31.7)		
Sensitivity (%)	39.6 (38.0, 41.2)			54.9 (52.8, 57.0)			64.8 (62.5, 67.1)		
Specificity (%)	91.5 (90.1, 92.9)			89.5 (88.3, 90.6)			87.2 (86.1, 88.4)		
PPV (%)	91.8 (90.4, 93.2)			80.9 (78.9, 82.9)			72.3 (70.0, 74.6)		
NPV (%)	38.7 (37.1, 40.4)			70.9 (69.4, 72.5)			82.8 (81.5, 84.1)		
Kappa (κ)	0.223 (0.205, 0.241)			0.458 (0.435, 0.482)			0.533 (0.508, 0.558)		

*TC* total cholesterol, *LDL-C* low density lipoprotein cholesterol, *OBJ+* positive objective measure, *OBJ–* negative objective measure, *SR+* positive self-reported measure, *SR–* negative self-reported measure, *PPV* positive predictive value, *NPV* negative predictive value

The overall agreement of self-reported HC with objective HC at the standard threshold was fair (κ = 0.223). Sensitivity was 39.6% and NPV were 38.7%, whilst specificity and PPV were higher at 91.5% and 91.8%, respectively. Sensitivity increased to 64.8% at the highest diagnostic threshold and consequently NPV also increased to 82.8%. Specificity remained >85% and PPV declined slightly to 72.3% at the highest threshold. Agreement increased to moderate levels (κ = 0.458 and 0.533, respectively) at the middle and highest diagnostic thresholds.

### Predictors of sensitivity and specificity of self-report

Table 4 shows that the variables which were independent predictors of higher (OR) sensitivity of self-reported HTN at the standard threshold were increasing age, increasing BMI, worse self-rated health, diabetes and family history of HTN. The following variables were not significant predictors of sensitivity of self-reported HTN or HC at any threshold in the initial model and so were excluded from the final model: marital status, income, highest level of education, smoking, excessive alcohol, physical activity and long-standing illness.

**Table 4:** Probability, by multivariable logistic regression, of subjects with objective hypertension or hypercholesterolaemia self-reporting ‘yes’ (sensitivity)

Variable	Hypertension			Hypercholesterolaemia		
	**≥140, ≥90, med.**^a)^	**≥150, ≥95, med.**	**≥160, ≥100, med.**	**≥5.5, ≥3.5, med.**^b)^	**≥6.2, ≥4.1, med.**	**≥6.5, ≥4.9, med.**
Age (linear)	1.04 (1.02, 1.06)^**^	1.02 (0.99, 1.04)	1.02 (0.99, 1.05)	1.05 (1.04, 1.06)^**^	1.04 (1.02, 1.05)^**^	1.03 (1.01, 1.05)^**^
Sex						
Male	1.00	1.00	1.00	1.00	1.00	1.00
Female	1.01 (0.84, 1.21)	0.95 (0.75, 1.20)	0.94 (0.69, 1.27)	0.88 (0.76, 1.01)	0.85 (0.71, 1.02)	0.75 (0.60, 0.93)^**^
BMI (linear)	1.03 (1.01, 1.05)^**^	1.03 (1.01, 1.06)^**^	1.06 (1.03, 1.10)^**^	1.01 (0.99, 1.02)		1.01 (0.99, 1.04)
Self-rated health						
Poor or fair	2.03 (1.32, 3.11)^**^	1.29 (0.73, 2.27)	1.41 (0.67, 2.95)	2.17 (1.54, 3.04)^**^	1.96 (1.31, 2.93)^**^	1.75 (1.09, 2.80)^*^
Good	1.53 (1.08, 2.16)^*^	0.99 (0.62 1.59)	0.90 (0.49, 1.67)	1.95 (1.52, 2.52)^**^	2.02 (1.49, 2.73)^**^	1.84 (1.28, 2.64)^**^
Very good	1.32 (0.93, 1.87)	1.00 (0.62, 1.61)	1.15 (0.61, 2.17)	1.36 (1.06, 1.76)^*^	1.38 (1.02, 1.86)^*^	1.42 (0.99, 2.04)
Excellent	1.00	1.00	1.00	1.00	1.00	1.00
History of CVD						
No	1.00	1.00	1.00	1.00	1.00	1.00
Yes	1.00 (0.71, 1.39)	0.62 (0.43, 0.89)^**^	0.37 (0.25, 0.56)^**^	2.15 (1.56, 2.96)^**^	1.53 (1.08, 2.17)^*^	1.14 (0.78, 1.65)
Diabetes mellitus						
No	1.00	1.00	1.00	1.00	1.00	1.00
Yes	1.58 (1.15, 2.18)^**^	1.39 (0.94, 2.06)	0.85 (0.55, 1.31)	3.76 (2.75, 5.13)^**^	3.06 (2.11, 4.42)^**^	2.67 (1.76, 4.05)^**^
Family history HTN						
No or don’t know	1.00	1.00	1.00	1.00	1.00	1.00
Yes	2.30 (1.90, 2.77)^**^	2.48 (1.96, 3.15)^**^	2.63 (1.95, 3.55)^**^	1.40 (1.21, 1.62)^**^	1.39 (1.16, 1.66)^**^	1.39 (1.12, 1.72)^**^

Data are presented as odds ratio (95% confidence interval). Significant differences are indicated as ^*^P < 0.05, ^**^P < 0.01

^a)^≥140, ≥90, med., systolic blood pressure ≥140 mmHg and/or diastolic blood pressure ≥90 mmHg and/or anti-hypertensive medication use; ^b)^≥5.5, ≥3.5, med., total cholesterol ≥5.5 mmol/L and/or low-density lipoprotein cholesterol ≥3.5 mmol/L and/or lipid-lowering medication use

*BMI* body mass index, *CVD* cardiovascular disease, *HTN* hypertension

Increasing BMI and family history of HTN were the only other two variables with significant positive associations with sensitivity at the higher thresholds. Interestingly, having a history of CVD was associated with a lower sensitivity at the middle and higher HTN thresholds (compared with the null group).

Table 4 shows that increasing age, worse self-rated health, history of CVD, diabetes and family history of HTN were also independent predictors of a higher sensitivity of self-reported HC at the standard diagnostic threshold. All remained significant predictors at the higher thresholds except a history of CVD. At the highest threshold, women were also less likely to report the presence of HC than men (OR, 0.75; 95% CI, 0.60–0.93).

Table 5 shows that increasing age, history of CVD and family history of HTN were independent predictors of lower (OR) specificity of self-reported HTN at the standard threshold. The following variables were not significant predictors of specificity of self-reported HTN or HC at any threshold in the initial model and so were excluded from the final model: sex, marital status, income, highest level of education, smoking, physical activity and long-standing illness. At the higher HTN thresholds, increasing BMI, worse self-rated health and diabetes additionally predicted lower specificity of self-report.

**Table 5:** Probability, by multivariable logistic regression, of subjects without objective hypertension or hypercholesterolaemia self-reporting ‘no’ (specificity)

Variable	Hypertension			Hypercholesterolaemia		
	**≥140, ≥90, med.**^**a)**^	**≥150, ≥95, med.**	**≥160, ≥100, med.**	**≥5.5, ≥3.5, med.**^**b)**^	**≥6.2, ≥4.1, med.**	**≥6.5, ≥4.9, med.**
Age (linear)	0.96 (0.93, 0.99)^**^	0.96 (0.93, 0.98)^**^	0.95 (0.93, 0.98)^**^	0.92 (0.89, 0.95)^**^	0.94 (0.92, 0.96)^**^	0.96 (0.94, 0.97)^**^
Excessive alcohol						
No	1.00	1.00	1.00	1.00	1.00	1.00
Yes	0.70 (0.47, 1.05)	0.66 (0.49, 0.88)^**^	0.78 (0.60, 1.02)	0.68 (0.45, 1.03)	0.78 (0.60, 1.03)	0.79 (0.63, 1.00)
BMI (linear)	0.96 (0.92, 1.00)	0.95 (0.93, 0.98)^**^	0.95 (0.93, 0.98)^**^	0.95 (0.91, 0.99)^*^	0.99 (0.96, 1.01)	0.99 (0.97, 1.01)
Self-rated health						
Poor or fair	0.65 (0.27, 1.55)	0.43 (0.22, 0.81)^**^	0.49 (0.28, 0.85)^*^	0.41 (0.17, 0.97)^*^	0.33 (0.19, 0.60)^**^	0.43 (0.26, 0.69)^**^
Good	0.70 (0.38, 1.30)	0.58 (0.35, 0.96)^*^	0.61 (0.39, 0.93)^*^	0.62 (0.30, 1.28)	0.52 (0.32, 0.84)^**^	0.57 (0.39, 0.83)^**^
Very good	0.78 (0.42, 1.44)	0.64 (0.39, 1.05)	0.74 (0.49, 1.14)	0.74 (0.36, 1.53)	0.66 (0.41, 1.06)	0.76 (0.52, 1.10)
Excellent	1.00	1.00	1.00	1.00	1.00	1.00
History of CVD						
No	1.00	1.00	1.00	1.00	1.00	1.00
Yes	0.31 (0.15, 0.67)^**^	0.39 (0.21, 0.71)^**^	0.47 (0.27, 0.83)^**^	0.41 (0.19, 0.88)^*^	0.61 (0.32, 1.17)	0.69 (0.38, 1.26)
Diabetes mellitus						
No	1.00	1.00	1.00	1.00	1.00	1.00
Yes	0.51 (0.23, 1.13)	0.48 (0.27, 0.84)^*^	0.49 (0.30, 0.81)^**^	0.46 (0.23, 0.94)^*^	0.39 (0.23, 0.66)^**^	0.48 (0.30, 0.77)^**^
Family history HTN						
No or don’t know	1.00	1.00	1.00	1.00	1.00	1.00
Yes	0.38 (0.26, 0.57)^**^	0.41 (0.31, 0.56)^**^	0.44 (0.34, 0.56)^**^	0.63 (0.43, 0.94)^*^	0.59 (0.46, 0.77)^**^	0.64 (0.51, 0.79)^**^

Data are presented as odds ratio (95% confidence interval). Significant differences are indicated as ^*^P < 0.05, ^**^P < 0.01

^a)^≥140, ≥90, med., systolic blood pressure ≥140 mmHg and/or diastolic blood pressure ≥90 mmHg and/or anti-hypertensive medication use; ^b)^≥5.5, ≥3.5, med., total cholesterol ≥5.5 mmol/L and/or low-density lipoprotein cholesterol ≥3.5 mmol/L and/or lipid-lowering medication use

*BMI* body mass index, *CVD* cardiovascular disease, *HTN* hypertension

Table 5 also shows that increasing age, BMI, poor self-rated health, history of CVD, diabetes and family history of HTN were significantly associated with a lower (OR) specificity of self-reported HC at the standard threshold. Age, self-rated health, diabetes and family history of HTN remained significant predictors at the higher thresholds.

## Discussion

At standard diagnostic thresholds, this study found that self-reported HTN and HC had low-moderate sensitivities (30–58%) and NPVs (39–75%) with high specificities (>90%), leading to significant under-estimates of objective prevalence of these risk factors in the population. Agreement between self-reported and objective data was also only moderate for HTN (κ = 0.564) and fair for HC (κ = 0.223). These findings are generally consistent with those in the literature [8–13]. The under-estimation and misclassification of objective status by self-report at standard diagnostic thresholds leads authors to recommend against its routine use for population surveillance.

At higher diagnostic thresholds, this study found large absolute increases in sensitivity and kappa agreement, with relatively small declines in specificity. This reflects findings of previous papers [8, 10–12, 14]. At the highest threshold for HTN, sensitivity (81.7%), specificity (91.3%) and kappa (0.717) were substantial. Authors thus suggest that self-reported HTN is a reasonable indicator of moderate-severe HTN (i.e., BP ≥160/100 mmHg and/or use of anti-hypertensive medications). This is a novel finding of this study. However, self-reported HC data should be used with caution at all thresholds because there was still only moderate agreement between self-reported and objective HC at the highest diagnostic threshold (TC ≥6.5, LDL-C ≥4.5, and/or use of lipid-lowering medication).

A lack of awareness of mild HTN (BP 140/90 to 160/100 mmHg) may help to explain these results [8, 14] For example, current Australian guidelines [31], which are in line with international guidelines [32], recommend that treatment decisions for HTN and HC are based on assessments of overall absolute CVD risk. Lifestyle advice is recommended for BP 140/90 to 160/100 mmHg, when the individual’s absolute 5-year CVD risk is <15%, whereas anti-hypertensive medications are recommended for persistent BP ≥160/100 mmHg, regardless of absolute risk levels [31]. HTN that does not require pharmacological treatment, for example, may affect communication of the diagnosis by the clinician or interpretation by the patient.

Limitations of objective data may also help to explain the results. Many studies, including our own, rely on BP and serum cholesterol measures taken in a single sitting, which are known to misclassify individual’s HTN or HC status due to intra-individual (biological) and temporal variability of single measures [33, 34]. BP measures taken in a single sitting tend to over-estimate objective prevalences [35], in part due to white-coat HTN, which can occur in approximately 10% of people based on office measurements [36]. Substantial reductions in misclassification is achieved by including at least one additional BP or cholesterol measurement taken at a different time point [33, 37, 38]. It is possible that marked improvements in agreement at higher thresholds are due, in part, to lesser misclassification by single BP measures at these thresholds.

The relatively poor agreement of self-reported HC at all thresholds may relate to a relative lack of awareness of HC in the population, as well as changing standards for HC diagnosis based on results of lipid-lowering trials and recommendation for therapy based on an individual’s absolute CVD risk rather than cholesterol level alone [31, 32]. Also measuring serum cholesterol requires a blood sample, which creates greater barriers to detection and screening for HC.

Participant characteristics that independently predicted higher sensitivity of self-reports included increasing age, increasing BMI, worse self-rated health, diabetes and family history of HTN, each of which are recognised CVD risk factors [2]. These reflect findings in the literature [8–15]. We also found in the same BHAS cohort that the prevalence of multimorbidity increased with factors including age, obesity, and family history of diabetes or CVD [39]. Hence, participants with these risk factors are likely to have increased health consciousness and healthcare monitoring including measurements of BP and cholesterol [10].

As expected, factors that predicted higher sensitivity (increasing age, history of diabetes, and family history of HTN) were generally the same as those that predicted lower specificity at the standard threshold. Participants in these sub-groups may assume that they have HTN and HC based on their age and risk factors and so provide false positive self-reports [19]. In addition, people with CVD are routinely prescribed renin-angiotensin system inhibitors and lipid-lowering drugs for secondary prevention even if their BP and cholesterol levels are not elevated at the standard threshold.

Participant characteristics that predicted higher sensitivity or specificity of self-report were investigated at standard along with more stringent diagnostic thresholds, which is a novel approach of this study. Factors that were significant predictors at the standard threshold were mostly also significant predictors at more stringent thresholds and directions of associations were the same. Variations in factors with significant associations between thresholds may be related to decreasing numbers of participants who met criteria for HTN or HC at higher diagnostic thresholds. Varying sensitivity or specificity of self-report between sub-groups leads to differential bias in prevalence estimates for some sub-groups [15] and based on some participant characteristics [10]. For example, in this study, increasing BMI is associated with higher sensitivity of self-report, and therefore overweight or obese participants would be over-represented amongst those with self-reported HTN. As suggested by Newell et al. [6], researchers relying on self-reported data should conduct a validation sub-study and adjust their overall findings, to take into account factors likely to affect the sensitivity and specificity of self-report in their population sample.

Interestingly, a history of CVD predicted worse sensitivity of self-reported HTN at the middle (OR, 0.62) and higher (OR, 0.37) thresholds, while there was no significant association at the standard threshold (OR, 1.00). The association may be potentially explained by the fact that people with established CVD are more likely to have BP levels at the middle and higher thresholds and more likely to be unaware of having HTN because their HTN is first noted at the time of CVD diagnosis and their awareness is of having CVD only.

Being female predicted lower sensitivity of self-reported HC at the highest threshold (OR, 0.75) compared with being male, reflecting findings in the study by Chun et al. [9] of Korean adults aged 50+ years [9]. Women attending primary healthcare services in Australia are also known to be less likely than men to have CVD risk factors measured, and younger women with high CVD risk are less likely to be prescribed preventive medications and hence may not report a HC level even if present [40].

Strengths of this study include having a large sample size of middle-aged adults (*n* = 4876), a high overall participation rate (62%), rigorous quality control of data collection [23] and medication data that is coded from participants’ lists of doctor-prescribed medications. The Busselton population is racially homogenous (>99% Western European) and confined to a specific geographic setting [23]. Yet, characteristics of the survey sample are similar to Australians in that age group at that time, using nationally representative data [30]. This study took a single measure of BP in the right arm following five minutes of rest, whereas at least two resting BP measures are recommended in a single exam for BP screening in the community [38]. Nevertheless our results are comparable with studies that investigated comparisons using objective BP data that was taken as an average of two or three measures in a single sitting [8–13]. Objective measures employed in this study do not account for those who had the condition in the past and no longer have it, which may contribute to false positive self-reports (<3% of the total sample at standard thresholds).

The finding that self-reported HTN and HC have low-moderate agreement with clinical objective data, at standard diagnostic thresholds, have implications for population health surveillance studies, which have largely relied on self-report in Australia and elsewhere [3–5]. For example, without accurate estimates of true prevalences, health systems are unable to optimise chronic disease management and prevention strategies, which may leave portions of the population at risk of developing CVD.

## Conclusions

A novel contribution is that self-reported HTN may be a more reasonable indicator of moderate-severe HTN. Therefore, self-reported HTN may be useful to detect those taking anti-hypertensive medications or those with BP≥160/100 mmHg. Future studies should investigate comparisons at more stringent thresholds, as well as research into more reliable gold standards (such as 24-hour ambulatory BP monitoring) [36], as this may lead to differences in interpretation about the utility of self-report. Authors also conclude that wherever possible, researchers using self-reported health information should take into account factors likely to affect the sensitivity and specificity of self-report in their population sample and include a validation subsample [6].

## Data Availability

The study uses de-identified participant data from the Busselton Population Medical Research Institute (BPMRI) database. Investigators may access BPMRI data if they apply to the Institute for the purpose of original scientific research (email address: busseltonhealthstudy@bpmri.org.au). All data relevant to this study are included in the article.

## Abbreviations

BHAS: Busselton Healthy Ageing Study
BMI: Body mass index
BP: Blood pressure
CI: Confidence intervals
CVD: Cardiovascular disease
DBP: Diastolic blood pressure
HC: Hypercholesterolaemia
HTN: Hypertension
LDL-C: Low density lipoprotein cholesterol, NPV:Negative predictive value
OR: Odds ratios
PPV: Positive predictive value
SBP: Systolic blood pressure
TC: Total cholesterol
